# Effects of Huanglian Jiedu Decoration in Rat Gingivitis

**DOI:** 10.1155/2018/8249013

**Published:** 2018-01-16

**Authors:** Fangbo Zhang, Ya Geng, Haiyu Zhao, Hongjie Wang, Yi Zhang, Defeng Li, Baolin Bian, Hongjun Yang

**Affiliations:** Institute of Chinese Materia Medica, China Academy of Chinese Medical Sciences, Beijing 100700, China

## Abstract

Gingivitis is an inflammatory disease that affects gingival tissues through a microbe-immune interaction. Huanglian Jiedu decoction (HLJD) is used traditionally for clearing and detoxifying in China, which had been reported to possess many pharmacological effects. Rat gingival inflammation model was established by lipopolysaccharide (LPS) injection for 3 consecutive days, and HLJD was given by gavage before LPS injection. After 3 days rats were sacrificed and tissue samples were evaluated. Serum cytokine levels such as interleukin-6 (IL-6) and tumor necrosis factor-*α* (TNF-*α*) were measured by enzyme-linked immunoabsorbent assay (ELISA). Oxidative stress related molecules such as total antioxidant capacity (T-AOC), malondialdehyde (MDA), and reactive oxygen species (ROS) were determined. Expression of AMP-activated protein kinase (AMPK) and extracellular signal-regulated kinases 1/2 (ERK1/2) signaling pathway were inspected by western blotting. Histological changes of gingival tissues were tested with hematoxylin-eosin (HE) staining. HLJD significantly decreased serum levels of IL-6 and TNF-*α*, suppressed generation of MDA and ROS, and enhanced T-AOC creation. Moreover, HLJD inhibited expressions of AMPK and ERK1/2. The inflammation severity of gingival tissue by HE staining was severe in model group but relieved in HLJD group obviously. HLJD exhibited protective effects against gingival damage through suppressing inflammation reaction and elevating antioxidation power.

## 1. Introduction

Gingivitis is the most common form of periodontal disease affecting 50% to 90% of the adults worldwide [1]. Gingivitis is a chronic inflammatory reaction caused by mainly gram-negative bacteria. It is mediated by upregulation of synthesis and release of various proinflammatory factors leading to excessive injury [2, 3]. Gingivitis could induce gingival bleeding, periodontal pocket formation, and destruction of connective tissue attachment surrounding the teeth [4]. Therefore, gingivitis prevention and treatment are important solutions to cure periodontitis.

Lipopolysaccharide (LPS) is an endotoxin generated by gram-negative bacteria, which influences lipoxygenase and cyclooxygenase inflammatory pathways and results in production of proinflammatory cytokines such as prostaglandin (PG), interleukin-1*β* (IL-1*β*), interleukin-6 (IL-6), interleukin-12 (IL-12), and tumor necrosis factor-*α* (TNF-*α*) [5]. These cytokines destroy periodontal tissue and alveolar bone, eventually leading to tooth loss. In conclusion, LPS plays a vital role in destroying gingiva, periodontal ligament, and alveolar bone through proinflammatory mediators [6]. In our research experimental animal model was established by LPS injection in rat gingivitis as reported [7, 8].

Oxidative stress is involved in the pathogenesis of a number of diseases including arthritis, stroke, Alzheimer's disease, Parkinson's disease, and periodontal diseases [9, 10]. Large amounts of reactive oxygen species (ROS) generated by oxidative stress may contribute to gingival damage through a variety of different mechanisms such as lipid peroxidation, DNA damage, protein damage, oxidation of important enzymes, and stimulation of proinflammatory cytokines [11, 12]. AMP-activated protein kinase (AMPK) and extracellular signal-regulated kinases (ERKs) are not only known to be activated by ROS in oxidative stress, but also studied widely in gingival experiment. They both play key roles in mediating cell survival and cell death [13].

Local and systemic antibiotics have also been widely used in the therapy of gingivitis, but only slight improvements have been noted in most cases, and it goes along with concern about increased antibiotic resistance [14]. Thus, therapeutic agents modulating inflammatory mediators show promise for adult periodontitis and may be very effective in persons at a higher risk for periodontitis [15]. However, novel anti-inflammatory compounds or natural products without side effects in gingivitis diseases remain to be exploited necessarily.

Huanglian Jiedu decoction (HLJD) consists of* rhizoma coptidis*,* radix scutellariae*,* cortex phellodendri,* and* fructus gardeniae*, which is used traditionally for clearing and detoxifying in China. HLJD had been reported to possess many pharmacological effects such as anti-inflammation, antihyperlipidemia, antiatherosclerosis, antilipid peroxidation, and cerebral protection [16–18]. In our study HLJD was firstly evaluated in rat gingivitis. metronidazole and fenbufen capsules (metronidazole 100 mg, fenbufen 75 mg, MFC) served as the control. Our research revealed that HLJD has a positive effect in rat gingivitis.

## 2. Materials and Methods

### 2.1. Experimental Animals

Male Sprague-Dawley rats were purchased from Beijing Vital River Laboratory Animal Technology Co., Ltd.; license Number was SCXK2006-0009, weight (200 ± 20) g. The study was conducted according to the local ethical guidelines for animal care and usage.

### 2.2. Preparation of HLJD Powder

*Rhizoma coptidis*,* radix scutellariae*,* cortex phellodendri,* and* fructus gardeniae* (weight ratio 3 : 2 : 2 : 3) were crushed into small pieces and mixed. The mixture was refluxed with water (1 : 10, w/v) for 2 h. The filtrates were collected, and the residues were then refluxed in water (1 : 10, w/v) for 2 h. Two batches of filtrates were combined. Afterwards the concentrated extract was dried by vacuum concentration to obtain HLJD extract [18].

### 2.3. Animal Model

LPS (Sigma, USA) were dissolved in 0.9% saline and final concentration was 1.0 mg/ml. Rats received intraperitoneal anesthesia by phenobarbital sodium (1%, 40 mg/kg). Then Rats were injected with 10 *μ*l LPS in submucosal gingivitis at 6 sites, including the left, middle, and right of the upper and lower labial aspect of the incisor teeth for 3 consecutive days. Insulin syringe needles (0.3 × 13.0 mm, BD, USA) were used for all injections.

### 2.4. Animal Treatment

Rats were randomly divided 7 groups with 10 animals in each group as follows: normal control group: rats were untreated and fed normally; saline control group: rats received 10 *μ*l saline injections in submucosal gingivitis under general anesthesia at 6 sites; model group: rats were injected with 10 *μ*l LPS (1.0 mg/ml) in submucosal gingivitis under general anesthesia at 6 sites; three HLJD groups: rats were given different concentrations of HLJD solution (0.25 g/kg, 0.50 g/kg, and 1.00 g/kg) by intragastric administration and then were injected with LPS as model group; positive control group: MFC (Permit number H13024465, Product Batch number 371141042, China Shijiazhuang Pharmaceutical Group Co., Ltd.) was used as a positive control. Rats were given MFC solution (metronidazole 31.25 mg/kg, fenbufen 7.80 mg/kg) intragastrically and then injected with LPS as model group. Animal experiment process lasted four days. Rats were given HLJD or MFC solution once daily and then anesthetized to inject LPS in submucosal gingivitis for 3 consecutive days. Rats were sacrificed to do a variety of experiments on the fourth day.

### 2.5. Histological Examination

An isolated piece of tissue taken from central incisor gingival area was fixed in 10% formalin solution. After histological processing, samples were embedded in paraffin and stained with hematoxylin-eosin (HE). Tissue slides were assayed by light microscopy. Histological grading was evaluated mainly by inflammatory changes such as inflammatory cells infiltration and collagen fiber bundles variation.

### 2.6. Serum Cytokine Detection

After rats were anesthetized, blood samples were collected by abdominal aortic method and then centrifuged (3,000 rpm, 10 min) to get serum. Serum proinflammatory cytokines including IL-6 and TNF-*α* were measured by ELISA kits (Wuhan Huamei, China) according to the manufacturer's specifications. Absorbance of each sample was measured with a microplate reader (Molecular Devices, USA) at a wavelength of 450 nm.

### 2.7. Biochemical Analysis

Rat periodontitis tissue samples were crushed by ultrasonic wave (Ningbo Xinzhi, China), and the lysates were resuspended. T-AOC and MDA were measured by a microplate reader (Molecular Devices, USA) in accordance with the protocol of the detection kit. Protein content was measured with BCA™ Bradford protein assay (Pierce, USA).

### 2.8. Intracellular ROS

Rat periodontitis tissue samples were crushed by ultrasonic wave (Ningbo Xinzhi, China), and centrifuged at 5,000 rpm/min for 5 min. The precipitate was incubated with 10 *μ*M DCFH-DA (Biyuntian Biotechnology, China) for 30 min and then washed three times with phosphate-buffered saline (PBS) to remove residual probe. The cellular fluorescence intensities were detected with a fluorescence microplate reader (Molecular Devices, USA). Excitation and emission wavelengths were set at 488 nm and 525 nm, respectively.

### 2.9. Western Blotting Analysis

Protein samples were performed by lysing rat periodontitis tissues in RIPA buffer containing protease inhibitors. Protein concentration was determined by BCA Bradford protein assay kit (Pierece, USA). Each protein sample was resolved by sodium dodecyl sulfate polyacrylamide gel electrophoresis (SDS-PAGE) and then transferred onto a PVDF membrane (Millipore, USA) electrophoretically. The membrane was blocked with 5% skim milk, sequentially incubated with primary antibody (Santa Cruz Biotechnology, USA) and secondary antibody (Zhongshanjinqiao, China) followed by electrochemiluminescence (ECL) detection (Shanghai Qinxiang, China). GAPDH (Cell Signaling Technology, USA) served as an internal control.

### 2.10. Statistical Analysis

Data were presented as means ± SD of at least three independent experiments. The significance of the differences among groups was compared by one-way ANOVA test. Values of *P* < 0.05 were considered to be statistically significant.

## 3. Results

### 3.1. HLJD Alleviates Rat Gingivitis Injury

**Figure 1:**
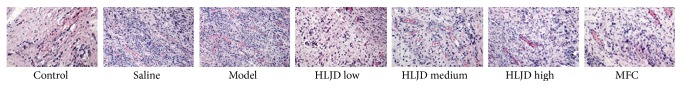
Histological comparison in rat gingiva tissue by HE staining (magnification ×200). Severe inflammation was caused in model group, and inflammation reaction was alleviated by HLDJ groups with a dose-effect mode.

Control group showed that epithelial cells proliferation was normal, and dense collagen fibers arranged regularly. In model group considerable inflammatory cells infiltrated in epithelial layer, and disrupted collagen fiber bundles were significantly observed in various directions. HLJD groups showed that slight inflammatory cells infiltration exited, and collagen fiber bundles were essentially reduced (Figure 1).

### 3.2. HLJD Decreases Serum Level of Inflammatory Cytokines

**Figure 2:**
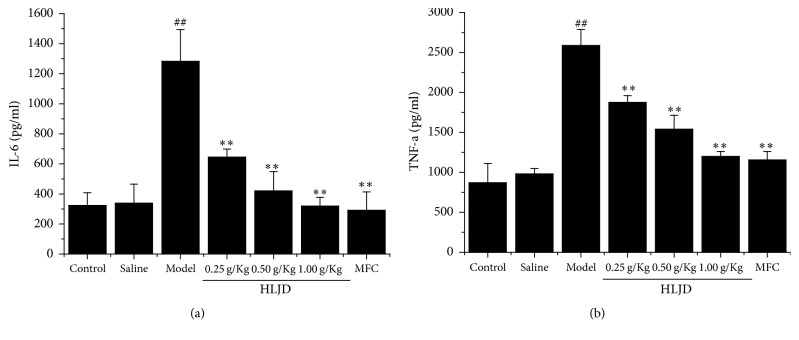
Serum inflammatory level of IL-6 (a) and TNF-*α* (b) in rat gingiva tissue by Elisa test. HLJD decreases serum level of inflammatory cytokines such as IL-6 (a) and TNF-*α*. ^##^*P* < 0.05, compared with control, and ^*∗∗*^*P* < 0.05, compared with model.

Serum inflammatory cytokines including IL-6 and TNF-*α* were analyzed by ELISA test. Level of IL-6 and TNF-*α* were increased remarkably in model group. In addition, level of IL-6 and TNF-*α* in three HLJD groups was clearly lower than those in model group, which showed an obvious dose-effect relationship (Figure 2).

### 3.3. HLJD Protected Rat Periodontitis Damage through Reducing Oxidative Stress

**Figure 3:**
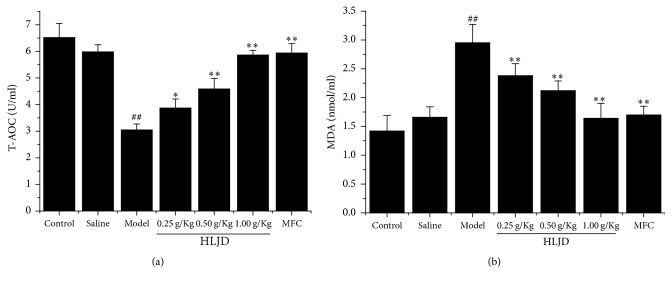
T-AOC (a) and MDA (b) level in rat gingiva tissue by biochemical assay kit. HLJD suppressed MDA generation and enhanced T-AOC creation. ^##^*P* < 0.05, compared with control, ^*∗*^*P* < 0.01, compared with model, and ^*∗∗*^*P* < 0.05, compared with model.

Oxidative stress has been implicated in the pathogenesis of inflammation. To determine whether HLJD affected oxidative stress related biochemical enzymes, T-AOC and MDA were measured in rat periodontitis lysates. Results showed that the activities of T-AOC were increased in HLJD group relative to model group, whereas MDA production was reduced in HLJD group compared with model group. Both T-AOC and MDA results were altered in a dose-dependent manner (Figure 3).

### 3.4. HLJD Attenuated Intracellular ROS Generation

**Figure 4:**
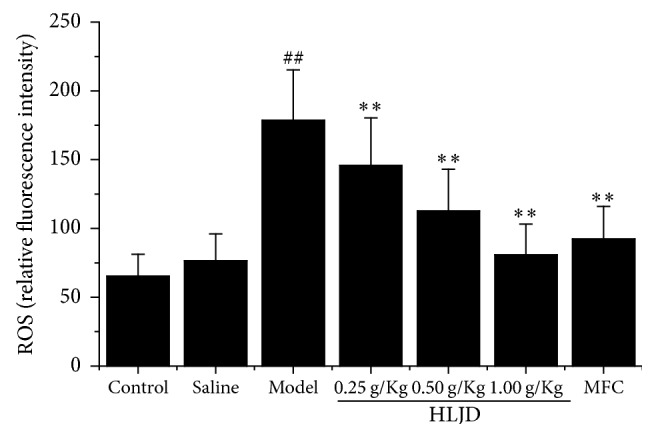
ROS level in in rat gingiva tissue by DCFDA assay. HLJD inhibited ROS production and reduced oxidative stress damage. ^##^*P* < 0.05, compared with control, and ^*∗∗*^*P* < 0.05, compared with model.

The generation of intracellular ROS by oxidative stress promotes tissue damage. Intracellular ROS were measured by DCFH-DA assay in accordance with the kit instruction. Intracellular ROS generation was obviously increased in model group. However, ROS generation was dramatically decreased by HLJD treatment in a concentration-related pattern (Figure 4).

### 3.5. HLJD Modulated AMPK and ERK1/2 Signaling Pathway Caused by LPS

**Figure 5:**
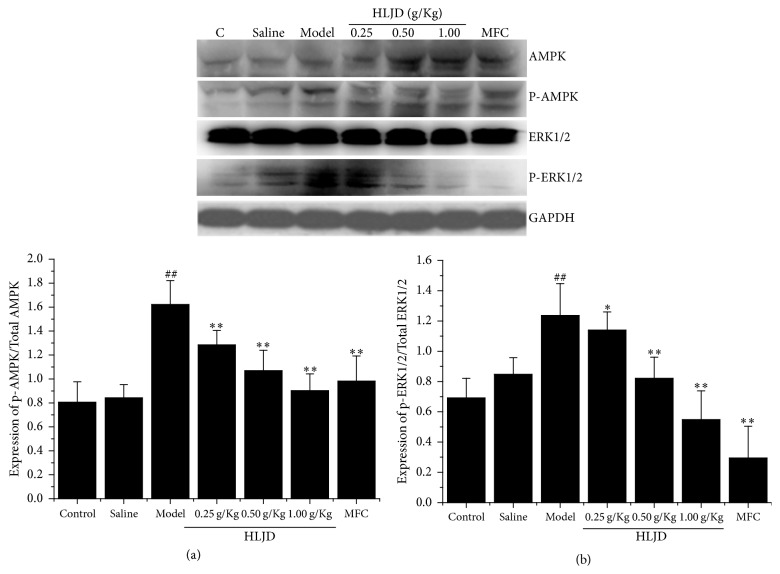
Expression of AMPK (a) and ERK1/2 (b) signaling pathway in rat gingiva tissue by western blotting. HLJD suppressed total phosphorylation expression of AMPK and ERK1/2. ^##^*P* < 0.05, compared with control, ^*∗∗*^*P* < 0.05, compared with model, and ^*∗*^*P* < 0.01, compared with model.

AMPK and ERK1/2 signaling pathways are known to regulate cellular apoptosis. Effects of HLJD on AMPK and ERK1/2 activation were examined, which were directly linked with phosphorylation state. Results indicated that expression of p-AMPK/AMPK and p-ERK1/2/ERK1/2 was elevated in model group while it was declined in HLJD group. Furthermore, HLJD suppressed total phosphorylation expression of AMPK and ERK1/2 in taken group with a dose-response relation (Figure 5).

## 4. Discussion

We demonstrated for the first time that HLJD decoration could be responsible for a protective effect against LPS-stimulated gingivitis in the current study. Our report showed that HLJD could inhibit rat periodontal tissue destruction through inhibiting gingival oxidative stress, modulating proinflammatory cytokines and stress related signaling transduction pathways.

In our research 10 *μ*g LPS at six sites per day was injected in rat periodontal tissues continuously for 3 days. Obvious inflammation response such as macroscopic signs was observed after injection for 3 days; thus this modeling method was proved to be valid by histological detection. HLJD had exact therapeutic effect in rat gingivitis, which was confirmed by histological examination.

Oxidative stress plays an important etiological role in a number of diseases including periodontitis, arthritis, heart disease, stroke, alcoholism, Alzheimer's disease, and Parkinson's disease. Oxidative stress causes excessive production and ROS accumulation leading to tissue damage. Excessive tissue oxidative damage induces impaired cellular function, contributing to periodontitis pathological progression. Periodontal injury in turn produces oxidative stress, and such reactions will further aggravate oxidative damage in the gingival tissues [19]. Tissue oxidative damage, impaired cellular function, and periodontitis progression thus seem to be a self-perpetuating process [20]. HLJD decoration could reduce oxidative stress and restrain damaged periodontitis through inhibiting ROS generation. Meanwhile, HLJD also suppressed generation of MDA and enhanced T-AOC creation.

ROS generation has a significant effect on inhibiting immune responses while mitochondrial ROS can trigger secretion of proinflammatory cytokines, which is a conservative defense mechanism of innate immune cells [21]. Human periodontal ligament (HPDL) cells were once stimulated by LPS in vitro and then produced proinflammatory mediators such as IL-1*β*, IL-6, IL-12, TNF-*α*, and prostaglandin (PG) [22]. Serum levels of proinflammatory cytokines such as IL-1*β*, IL-6, and TNF-*α* were increased in a rat ligature-induced periodontitis model [12]. Our study demonstrated that serum levels of IL-6 and TNF-*α* in HLJD group were significantly lower than those in model group by LPS injection. Our results were consistent with previous findings.

AMPK is an energy-sensing enzyme for the regulation of multiple metabolic processes, activated in response to a reduction in cellular energy charge and a decrease in the ATP to AMP ratios. Studies have shown that AMPK pathway can be activated by oxidative stress [23]. AMPK has a dual function in gingival experiment, for example, lindenenyl acetate exposure upregulated the levels of phosphorylated AMPK in LPS-stimulated human periodontal ligament cells model [22]; however, genipin suppressed AMPK phosphorylation in TNF-*α* stimulated human periodontal ligament cells [13]. As one MAPK family member, ERK1/2 has been known to be involved in oxidative stress mediating cellular function including proliferation, differentiation, and transformation [23]. Genipin could suppress ERK1/2 phosphorylation in TNF-*α*-stimulated HPDLCs. Meanwhile ERK1/2 activation may promote inflammation and result in necrosis by upregulating IL-1 [13]. Our study showed that HLJD boosted antioxidation and anti-inflammatory ability by inhibiting AMPK and ERK1/2 expression.

## 5. Conclusions

**Figure 6:**
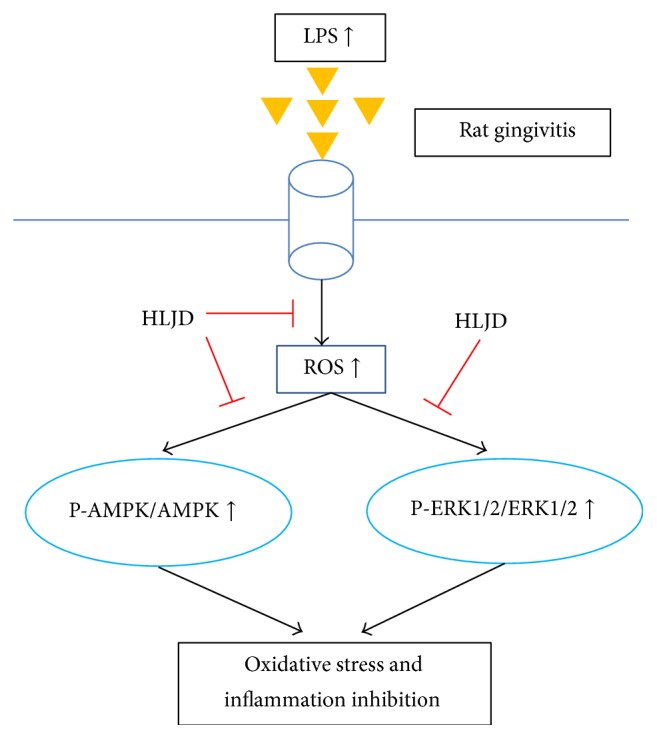
Schematic representation of HLJD inhibiting oxidative stress and inflammation by LPS in rat gingivitis.

HLJD obviously exhibited anti-inflammatory and antioxidant ability through decreasing serum levels of IL-6 and TNF-*α*, suppressing generation of MDA and ROS and enhancing T-AOC creation. Furthermore, HLJD suppressed AMPK and ERK1/2 expression. Thus HLJD could play a key role in the treatment of rat gingivitis (Figure 6).
